# Incidence and short term outcomes of neonates with hypoxic ischemic encephalopathy in a Peri Urban teaching hospital, Uganda: a prospective cohort study

**DOI:** 10.1186/s40748-018-0074-4

**Published:** 2018-03-07

**Authors:** Hellen Namusoke, Maria Musoke Nannyonga, Robert Ssebunya, Victoria Kirabira Nakibuuka, Edison Mworozi

**Affiliations:** 1 Department of Paediatrics and Child Health, Bethany Women and Family Hospital, P.O box 32022, Clock Tower, Kampala, Uganda; 2 Department of Paediatrics and Child Health, Mother Kevin Post Graduate Medical School, Uganda Martyrs University, Nkozi, Uganda; 3 Department of Paediatrics and Child Health, Makerere University and Mother Kevin Post Graduate School: Uganda Martyrs University, Nkozi, Uganda

**Keywords:** Arterial blood gases, Hypoxic ischemic encephalopathy, Intrapartum asphyxia, Newborn, Short term outcomes

## Abstract

**Background:**

Hypoxic Ischemic Encephalopathy carries high case fatality rates ranging between 10–60%, with 25% of survivors have an adverse long-term neurodevelopment outcome. Despite the above, there is paucity of data regarding its magnitude and short term outcomes in a low resource setting like Uganda. Therefore we set out to determine the incidence and short term outcomes of Newborns with Hypoxic Ischemic Encephalopathy at St.Francis Hospital, Nsambya.

**Methods:**

This was a Prospective Cohort study conducted between October 2015 and January 2016 at St. Francis Hospital, Nsambya, Kampala- Uganda. Term Newborn babies were enrolled. Umbilical cord arterial blood gas analysis was done for Newborns with low Apgar scores at 5 min. Clinical examination was done on all newborns within 48 h of life, for features of encephalopathy. Neonates with Hypoxic Ischemic Encephalopathy were followed up by a daily clinical examination and a short term outcome was recorded on day seven.

**Results:**

The incidence of Hypoxic Ischemic Encephalopathy was 30.6 cases per 1000 live births. The majority, 10 (43.5%) had mild Hypoxic Ischemic Encephalopathy, followed by 8 (34.8%), 5 (21.7%) that had moderate and severe Hypoxic Ischemic Encephalopathy respectively. A total of (6) 26% died, and (15) 65.2% were discharged within 1 week. Lack of a nutritive suckling reflex (nasogastric feeding), poor Moro reflex, and requirement for respiratory support (oxygen therapy by nasal prongs) were the common complications by day seven.

**Conclusions:**

The burden of Hypoxic Ischemic Encephalopathy is high with a case fatality rate of 26%**.** There is need to conduct a longitudinal study to determine the long term complications of HIE.

## Background

Globally, neonatal mortality accounts for up to 44% of the Under Five Mortality, of which 99% occurs in low and middle income countries [1, 2]. Intrapartum asphyxia and consequential Hypoxic Ischemic Encephalopathy (HIE) is a common cause of potentially avoidable neonatal brain injury and mortality [3]. Birth asphyxia and its complications is the third commonest cause, and contributes to 23% of neonatal mortality in Uganda [4].

Different measures are being taken to reduce neonatal mortality and morbidity. These include preventive measures such as; proper monitoring of labor with a partograph, timely and adequate resuscitation and therapeutic hypothermia of Newborns with HIE to improve outcome [5].

The incidence of HIE is 1.5 per 1000 live births in developed countries and varies between 2.3-26.5 per 1000 live births in developing countries [6, 7]. However, the incidence, and outcomes of HIE is not well documented in most developing countries, including Uganda. Therefore, this study is aimed at determining the incidence, and short term outcomes of HIE among inborn term neonates at a tertiary hospital.

## Methods

The study was conducted at St. Francis Hospital, Nsambya, a tertiary hospital providing Neonatal Intensive Care to Kampala and the surrounding districts. The standard of care for HIE at this unit included; Continuous Positive Airway Pressure(CPAP) for moderate and severe HIE to maintain respiratory support, intravenous Phenobarbital and Phenytoin to control seizures as first line and second line therapy respectively. Head cooling, parental nutrition, electroencephalogram (EEG) monitoring and mechanical ventilation were not a standard of care.

This was a prospective cohort study carried out on the labor ward and Neonatal unit of St. Francis Hospital, Nsambya from October 2015 to January 2016.

### Inclusion and exclusion criteria

All the following criteria (A + B + C) were required for inclusion in the study:

A. Term newborns delivered at ≥37completed weeks of gestation,
B. Birth weight > 2000 g
C. Delivery at St. Francis Hospital, Nsambya.

Newborns delivered by caesarian section under general anesthesia and those with congenital abnormalities were excluded.

### Sample size calculation:

A sample size of 679 participants was calculated for incidence of HIE using a formula for single proportions [8]. This was based on incidence of HIE of 1.8% in Mulago Hospital (similar setting) by Ondoa et al., allowing an error of 1% and confidence interval of 95% [9].

### Procedure

At delivery, Apgar score was done at one and 5 min by the attending midwife / research assistant.

A cord arterial blood sample was taken aseptically within 1 hour of delivery for newborns with an Apgar score of less than 7 at 5 min [10]. A heparinized needle was used, and at least 0.2 ml of arterial blood for blood gas analysis was drawn from the umbilical artery. Blood gas analysis was performed using ABL 80 FLEX analyzer within 5 min of sample collection. Clinical examination was done on all newborns within 48 h of life, to assess for features of encephalopathy. These included change in level of consciousness, altered primitive reflexes, tone and autonomic response.

Blood samples for Random Blood Sugar (RBS), C-reactive protein (CRP) were aseptically drawn from the peripheral veins, to rule out hypoglycemia and infection respectively.

All newborns with low APGAR scores (less than 7 at 5 min), intrapartum asphyxia (PH < 7.0, or base deficit ≥12.0 mmol/l) and features of encephalopathy were diagnosed to have HIE. Newborns with HIE were classified into mild, moderate and severe forms using the Modified Sarnat Encephalopathy Grading system (MSEG) as defined by Shalak et al. [11].

Neonates with HIE were managed according to standard operating systems at the unit. These were followed up by a daily clinical examination and short term outcome was recorded on day seven. The short term outcomes of interest included; death, alive without complications or alive with complications (requirement of respiratory support, absence of a nutritive suckling reflex, altered level of consciousness, presence of seizures, altered Moro reflex).

### Data management

Coded Data was entered using EPI-DATA version 3.1, and STATA version 11 was used for Statistical analysis. The Chi-square test was used to compare categorical variables and a student t-test for continuous variables with a 95% confidence interval (CI). A P -value of less than 0.05 was significant.

Factors that were statistically significant at bivariate level, and those that are known to be strongly associated with HIE like non-use of partograph to monitor labor, were further analyzed at Multivariate level. This was done using logistic regression fitting models to determine the factors that are statistically and independently associated with HIE. The factors with an adjusted Odds ratio of greater than one and a *P*-value of < 0.05 were considered statistically significant.

## Results

During the study period, 751 newborns were enrolled and 573 newborns excluded (140 were preterm, 32 were still births, 1 had a caesarian delivery under general anesthesia, and 400 did not consent for the study). There were 399 (53%) male and 352 (47%) female participants. The characteristics of the infants and their mothers are shown in Table 1.

**Table 1:** Summary of Maternal and Newborn demographics at St. Francis Hospital that participated in the study

	Frequency(*n* = 751)	Percentage
Sex of the baby		
*Male*	399	53
*Female*	352	47
Baby weight		
*< 2.5 kg*	14	2
*> =2.5 kg*	737	98
Age of the mother		
*< 20*	17	2
*20-35*	634	84
*> =35*	100	13
Distance from place of residence to Nsambya Hospital in KM		
*< =5*	244	32
*5-10*	237	32
*> 10*	270	36
Referral case		
*Yes*	26	4
*No*	712	96
Fetal heart rate		
< =120	28	4
> 120	723	96
APGAR score at 1 min		
< 7	58	8
> =7	693	92
APGAR score at 5 min		
< 7	25	3
> =7	726	97
Resuscitation done		
Yes	79	11
No	629	89

### Laboratory results for the participants

From this study, 25/751 (3.3%) newborns had low APGAR scores and intrapartum asphyxia as evidenced by either PH < 7.0 or Base deficit of ≥12.0. Two Newborns with intrapartum asphyxia had hypoglycemia (RBS < 2.5Mmol/l), and infection (CRP > 10 mg/dl).

### Incidence of HIE

**Fig. 1:**
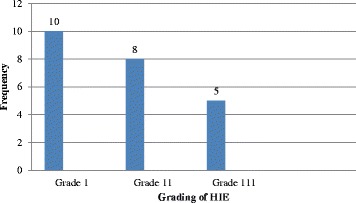
A bar graph showing the distribution of participants with HIE according to MSEG system at St. Francis Hospital, Nsambya

Of the 751 Newborns enrolled, 58 (8%), and 25 (3%) had low Apgar scores at one and 5 min respectively. All the newborns with low Apgar scores at 5 min had intrapartum asphyxia (as evidenced by low Apgar score, and either PH < 7.0 or Base deficit ≥12.0 mmol/l). A significant proportion of newborns with intrapartum asphyxia [23 of the 25(92%)] got HIE. The proportion of HIE in this study was found to be 3% (24/751); (95% CL: 1.8-4.2*).* Therefore the incidence of HIE was 30.6 cases per 1000 live births. In this study, 10 (43.5%), 8(34.8%), and 5 (21.7%) had grade I, grade 11 and grade III HIE respectively as shown in Fig. 1.

### Factors associated with HIE

The factors that were significantly associated with HIE at Bivariate analysis included: herbal medicine use (COR-3.2, *P* = 0.004), prolonged labour (COR-3.9, P = < 0.001), proloned rupture of membranes (COR-2.6, *P* = 0.016), Referred mothers (COR-3.9, *P* = 0.036), antepartum haemorrhage (COR-4.37, *P* = 0.024) prolonged pregnancy (COR-2.46*, P* = 0.025), Ceasarian delivery (COR-2.3, *P* = 0.035) and prime parity (COR-2.46, P = 0.025). On the contrary, factors like maternal illness, prolonged pregnancy, and non use of partograph were not significantly associated with HIE in this study as shown in Table 2.

**Table 2:** Bivariate table showing factors associated with HIE at St. Francis Hospital, Nsambya

	No HIE *n* = 728	HIE *N* = 23	COR(95% C I)	*p*-value
History of Herbal Use				
*No*	534(73)	11(48)	1	
*Yes*	194(27)	12(52)	3.190(1.4-7.2)	0.006
Referral				
*No*	705(97)	20(87)		
*Yes*	23(3)	3(13)	3.907(1.1-14.0)	0.036
Antepartum hemorrhage				
*No*	707(97)	20(87)	1	
*Yes*	21(3)	3(13)	4.373(1.2-15.7)	0.024
Prime parity				
*No*	535(74)	13(57)	1	
*Yes*	188(26)	10(43)	2.461(1.1-5.4)	0.025
Illness during pregnancy				
*No*	512(70)	20(87)	1	
*Yes*	216(30)	3(13)	0.301(0.1-1.0)	0.052
Prolonged pregnancy				
*No*	631(86)	18(78)	1	
*Yes*	97(14)	5(22)	1.527(0.6-4.1)	0.406
Duration of labor in hours				
*< =10*	564(77)	9(39)	1	
*> 10*	164(23)	14(61)	3.991(1.8-8.8)	0.001
Was a partograph used				
*No*	175(24)	9(39)	1	
*Yes*	553(76)	14(61)	0.479(0.2-1.1)	0.075
Fetal heart rate				
*< =120*	24(3)	4(18)	1	
*> 120*	704(97)	19(82)	0.188(0.1-0.6)	0.004
Prolonged rupture of membranes				
*No*	450(62)	9(39)	1	
*Yes*	278(38)	14(61)	2.603(1.2-5.8)	0.02
Mode of delivery				
Normal	508(70)	12(52)	1	
c-section	220(30)	11(48)	2.326(1.1-5.1)	0.035

Infants born to mothers with; Antepartum haemorrhage, history of herbal use, refferal and prime parity were significantly associated with HIE at logistic regression as shown in Table 3.

**Table 3:** multivariate table showing factors that are independently associated with HIE between at St. Francis Hospital, Nsambya

	COR	*P* –value	AOR	95% confidence Interval	*p*-value
Referral case	3.9	0.036	4.058	(1.0-15.7)	0.043
Antepartum hemorrhage	4.373	0.024	5.215	(1.0-26.2)	0.045
Prime parity	2.461	0.025	3.242	(1.3-7.9)	0.01
Illness during pregnancy	0.301	0.052	0.248	(0.1-0.9)	0.031
History of Herbal Use	3.190	0.006	5.291	(2.1-13.4)	0.000
Duration of labor					
*> 10 h*	3.991	0.001	14.867	(0.8-265.2)	0.066
Use of partograph	0.479	0.075	0.033	(0.0-1.0)	0.048
Caesarian delivery	2.326	0.035	0.091	0.0-161.3	0.529

*COR* Denotes for Crude Odds Ratio, *AOR* Denotes for Adjusted Odds Ratio

### Short term outcome of HIE

**Fig. 2:**
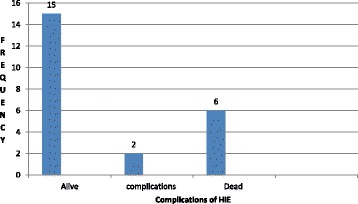
Bar graph showing surmary of short term complications of Hypoxic Ischemic Encephalopathy at St.Francis Hospital, Nsambya

Of the 23 participants who got HIE, 6/23 (26%) died. Two thirds (4/6) had grade III HIE, and the rest (2/6) had grade II HIE. The majority, 15/23 (65.2%), were discharged without short term complications, while 2/23 (8.6%) were still admitted with complications by day seven. The complications included: poor Moro reflex, lack of a nutritive suckling reflex (nasogastric feeding), and respiratory support (oxygen therapy by nasal prongs). There were 7/23 (30%) with seizures and these were controlled by the fifth day. A surmmary of short term complications of HIE is shown in Fig. 2.

## Discussion

### Incidence of hypoxic ischemic encephalopathy

The incidence of Hypoxic Ischemic Encephalopathy of 30.6 cases per 1000 live births among Newborn babies delivered at St. Francis Hospital, Nsambya, Uganda is still high, despite a number of interventions in place. This finding is similar to that reported in developing countries of 2.3-26.5 per 1000 live births by Kurinczuk JJ et al. [6].

This is contraly to the incidence of 39 cases per 1000 live births reported by Chiabi and collegues in Cameroon [12]. There is a high number of refferal cases in Chiabi et al. study of 46.7 and 15.6% from health centres and other hospitals respectively versus 4% found in our study [12]. Therefore there was a high number of complicated deliveries in Cameroon contributing to a high incidence of HIE.

The incidence of HIE in this study was higher than that reported in South Africa (8.5-13.3 per 1000 live births) and India (19.97 cases per 1000 live births) [13, 14]. The lower incidence in South Africa could have been attributed to better monitoring equipment of mothers during labour like cardiotopograph (helps in early detection of fetal distress for early intervention) which are lacking in the setting.

### Factors associated with HIE

Maternal factors such as prime parity, antepartum haemorrhage, referred mother and history of herbal use during labour were significantly associated with HIE.

Prime parity was a significant and common factor associated with HIE in several studies done in Uganda, India, and Saudi Arabia [9, 14, 15]. First time mothers are more predisposed to most antenatal-related-obstetric complications compared to multiparous mothers, and therefore caution should be taken in close monitoring and management of the prime gravidas to prevent complications [16].

On the contrary, factors like prolonged labour, prolonged pregnancy, maternal illness and delayed time between decision making and delivery were significantly associated with HIE in similar studies in Nigeria [17], which was not the case in our study. This could be attributed to the low numbers of mothers noted with the above factors in our study.

Studies by Bikisu et al. in Nigeria and Queresh et al. in Abbottabad [18, 19] showed that abscence of examination and not attending antenatal care were associated with HIE. Similar observations were made in our study where absence of monitoring of labour and being born to a refered mother was associated with HIE.

In addition, use of herbal medicine during pregnancy and labour was significantly associated with HIE (OR-5.2: CL:2.1-13.4, *P* = 0.000). This was however not documented in other studies, probably because it may not be the social practice among mothers in other settings.

### Short term outcome of hypoxic ischemic encephalopathy

The study established that 26% of the study participants that got HIE died, of which two thirds was due to grade III HIE. This was similar to a mortality of 26.4% in a prevalence study by Athumani et al. in Muhimbili, Tanzania [20].

These findings slightly differ with a report from a multicenter study by Massaro et al., where 945 Newborns with HIE from different Neonatal Intesive Care Units (NICU) in Washington D.C were followed up for 2 weeks. In this study, 15% of participants died, 85% had complete feeding by discharge, 7.6% required nasogastric feeding, and 6% required supplemental oxygen therapy with < 1% that required tracheostomy [21]. The lower mortality could be explained by the use of therapeutic hypothermia that has been proven to significantly reduce morbidity and mortality from earlier studies [22]. The advanced newborn care in a resourceful setting like the US, was not a practice in this study.

In a South African study, a mortality of 7.8% was reported. This was much lower than that found in our study, though most of the deaths were due to grade three HIE as was the case in our study [13]. Therapeutic cooling was used as part of the management for those with moderate and severe HIE with better monitoring equipement which was not the case in our study. This may be a contributing factor to the low mortality in this study.

The mortality of 26% noted in this study was higher than that (12.1%) reported in a similar study done in Mulago Hospital in Kampala Uganda [9]. This variation could have been as a result of the lower follow up time of 48 h in a study by Ondoa et al. as compared to the 1 week used for this study.

The level of mortality was slightly lower than that reported in a similar study in India of 31.14% [14]. This may be because the study was carried out during the initiation phase of our neonatal intensive care unit. This has substantially promoted better nursing care.

### Strength and limitations

Arterial blood gases were done to confirm evidence of metabolic acidosis in the diagnosis of Hypoxic Ischemic Encephalopathy. However, we are mindful of some limitations like our inability to perform Cranial scans to rule out other possible causes of encephalopathy such as CNS malformations. We were as well unable to do blood cultures to rule out neonatal infections (we used a crp of > 10 mg/dl to diagnose sepsis among participants).This may have contributed to the high incidence of HIE noted. Additionally, there were low numbers of Newborns with HIE which could limit generalisability.

## Conclusion

The incidence of Hypoxic Ischemic Encephalopathy at St. Francis Hospital, Nsambya was high at 30.6 cases per 1000 live births. Majority (17/23 (73.9%)) of newborns with Hypoxic Ischemic Encephalopathy were discharged without short term complications by 1 week.

### Recommendations

A longitudinal study should be carried out to determine the long term complications of HIE.

## Abbreviations

AOR: Adjusted odds ratio
CI: Confidence interval
CNS: Central nervous system
COR: Crude odds ratio
CRP: C- reactive protein
CT: Computerized tomography
EEG: Electroencephalogram
HIE: Hypoxic ischemic encephalopathy
MSEG: Modified Sarnat encephalopathy grading
NICU: Neonatal intensive care unit
RBS: Random blood sugar
WHO: World health organization
